# Single cell whole genome sequencing reveals that NFKB1 mutation affects radiotherapy sensitivity in cervical cancer

**DOI:** 10.18632/oncotarget.23587

**Published:** 2017-12-21

**Authors:** Dong Yang, Weiyuan Zhang, JunQing Liang, Kexin Ma, Peng Chen, Danni Lu, Wenjing Hao

**Affiliations:** ^1^ Beijing Obstetrics and Gynecology Hospital, Capital Medical University, Beijing 100026, China; ^2^ Peking University People’s Hospital, Beijing 100044, China

**Keywords:** cervical cancer, radiotherapy, single cell sequencing, somatic mutation, gene

## Abstract

Cervical cancer is the third most common cancer in women. Radiotherapy resistance remains a major obstacle for patients with cervical cancer. Somatic alterations in human genomes are responsible for radiotherapy resistance. Here, we performed single cell whole genome sequencing on 13 cells before radiotherapy and 12 cells after radiotherapy from a Chinese woman patient with cervical carcinoma. We identified one damaging mutation in NFKB1 (G430E), which showed significantly increased mutant allele frequency after radiotherapy than that before radiotherapy. Further functional assays showed that NFKB1 was a tumour suppressor in cervical cancer by inhibiting cell proliferation, colony formation and migration, while the mutation in NFKB1 could weaken the tumour suppressing functions of NFKB1. NFKB1 enhanced the sensitivity of cervical cancer cells to the effects of irradiation, and the mutation in NFKB1 weakened this effect. These results suggested that NFKB1 may be a potential molecular target in cervical cancer radiation therapy in the future.

## INTRODUCTION

Cervical cancer is the third most common cancer in women with about 500,000 new cases each year, accounting for 5% of all new cancer cases [1]. The main cause of cervical cancer is persistent infection with oncogenic types of human papillomavirus (HPV) [2], which is detected in 99.7% of cervical cancer cases [3]. Although the treatment for cervical cancer have been largely successful in developed countries, most women in developing countries diagnosed with cervical cancer at advanced stages [4]. Therefore, cervical cancer is still the second most common cause of cancer-related deaths in women in developing countries [4]. Developing effective therapies for cervical cancer are urgently needed.

Radiotherapy is one of the most important treatments for cervical cancer [5]. Radiotherapy is used to treat up to 50% of cancer patients and to manage 40% of patients who are cured [6]. With the widespread use of precise radiotherapies such as conformal radiotherapy, intensity modulated radiotherapy, and image guarded radiation therapy, and the application of protons and heavy ions in the clinic, the role of radiotherapy has become increasingly important [5]. However, in clinical practice, most radiation practitioners have found that not every cancer patient responds to the treatment [7, 8]. Moreover, some patients only show good responses at the beginning of treatment [7, 8]. Somatic alterations in human genomes are responsible for radiotherapy resistance [9, 10]. For example, among cervical cancer patients who receive radiation therapy, patients harboring KRAS mutations have significant worse outcomes [11]. Previous reports have highlighted intratumoral heterogeneity in primary tumours, that is, the tumour cells from the same tumour harbor different somatic mutations, and cluster into different tumour subpopulations. As long as a small subpopulation of the tumour cells harbor somatic mutations which were responsible for radiotherapy resistance, the tumour may not be treated from radiotherapy. Thus, intratumoural heterogeneity and subpopulation diversity of cancer cells may be responsible for the phenomenon that patients only show good responses at the beginning of treatment [12, 13]. Precise understanding of intratumoural heterogeneity of cancer is crucial for the radiotherapy resistance for cancer.

Recently, several large-scale genomic studies have characterized cervical cancer genomes as having hundreds of somatic mutations in various genes, including *PIK3CA*, *PTEN* and *TP53* [14, 15]. Targeted therapy against specific somatic mutations in these genes have transformed the management of various cancers [16], and discovery of these candidate novel therapeutic targets in cervical cancer could develop new accurate therapies for cervical cancer. However, genomic alterations identified in all of these studies were obtained using only single samples representing individual cases, and little is known about the spatial intratumoural heterogeneity.

Single cell sequencing technology is demonstrated to be effective in investigating intratumoural heterogeneity in tumours [17, 18]. In view of the power of single cell sequencing technology, we performed single cell whole genome sequencing on 25 cells in tumour tissues from a Chinese woman patient with HPV-related cervical carcinoma classified as the IIA2 stage. This patient received radiation therapy (10 Gy). Among these cells, 13 cells were from tumour tissues before radiotherapy, and 12 cells from tumour tissues after radiotherapy. We found that a somatic missense mutation (G430E) in NFKB1 showed significant increased mutant allele frequency in tumour cells after radiotherapy. We further demonstrated that this mutation (G430E) could weaken the tumour suppressing functions of NFKB1 and could promote the survival of cervical cancer cells following irradiation. Our results provide an important molecular foundation of tumorigenesis and progression in radiation therapy of cervical cancer.

## RESULTS

### High throughput isolation and amplification of single cells from fresh tumour tissues

Fresh tumour tissues before and after radiotherapy were obtained from a 46-year-old Chinese woman with HPV-related cervical carcinoma classified as the IIA2 stage. The HPV type was detected as HPV 16 using flow-through hybridization. We also collected blood from this patient, which was used as the matched normal control. To obtain detailed cellular genetic information on this tumour, we carried out single cell sequencing in individual cells from the tumour samples as described previously [18]. We carried out whole genome amplification based on multiple displacement amplification of the DNA from each single cell of the tumour tissues (Supplementary Methods). In total, we obtained 13 cells from tumour tissues before radiotherapy, and 12 cells from tumour tissues after radiotherapy.

We performed massively parallel single cell whole genome sequencing on these samples using paired-end 150 bp reads. The blood sample also underwent con-ventional whole genome sequencing (WGS). Each sample achieved 113.9 Gb data, and the average sequencing depth was ∼38× (Supplementary Table 1). We then detected the somatic mutations for each cell using VarScan (v2.3.9) (MATERIALS AND METHODS). We only focused on mutations in coding regions and splice site regions. In total, we identified 139 somatic mutations (Supplementary Table 2).

**Figure 1:**
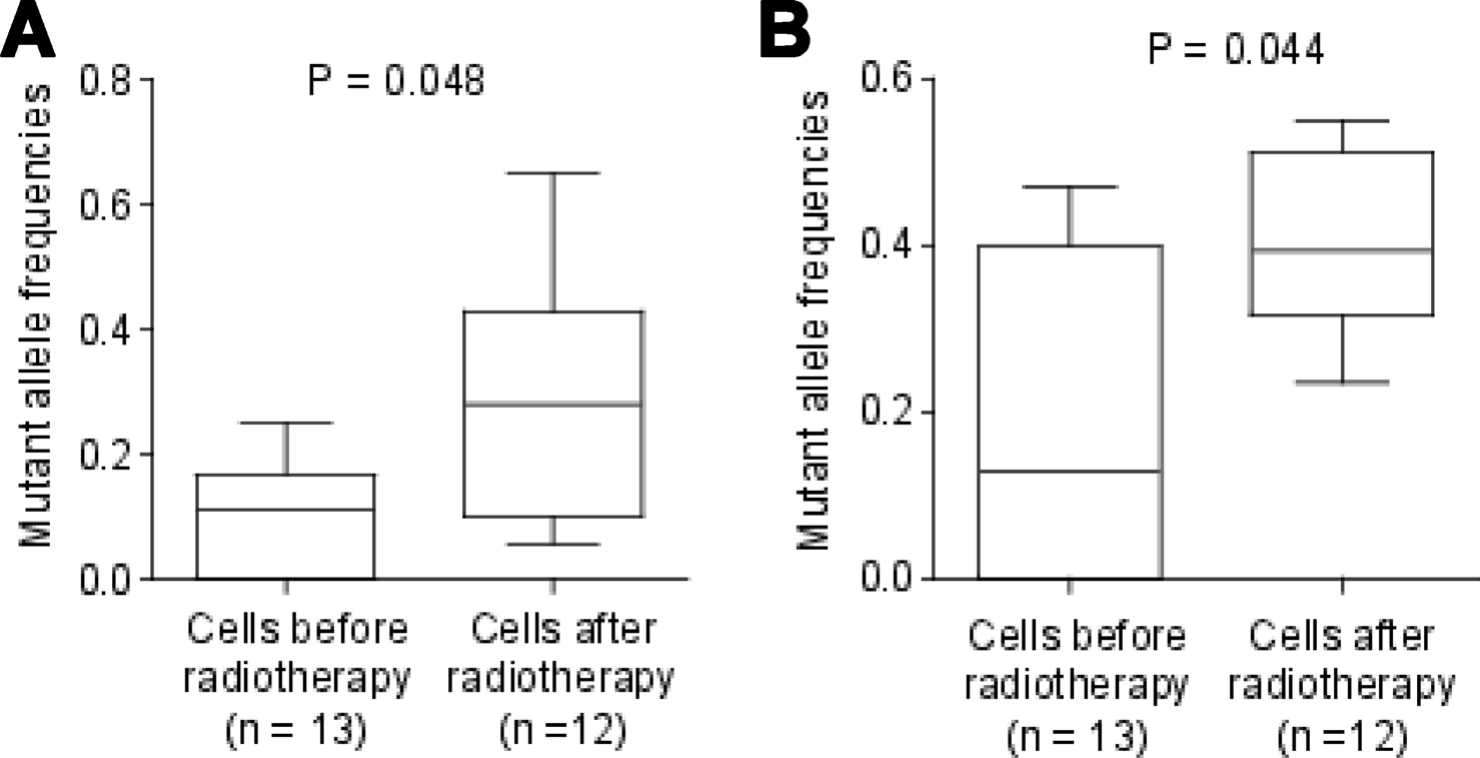
Mutant allele frequency for mutations in NFKB1 and AKAP6 Thirteen cells were from tumour tissues before radiotherapy, and 12 cells from tumour tissues after radiotherapy. Mutant allele frequencies for the mutations in NFKB1 (**A**) and AKAP6 (**B**) were shown. The *P* value was calculated using student *t* test.

We investigated the clonal status of cervical cancer cells before and after radiation therapy. The cancer cell fraction for tumours before and after radiation therapy was calculated as the mutation allele frequency of each cell before and after radiation therapy. In order to identify the mutations whose mutant allele frequencies were significantly increased in the cervical cancer cells after radiation therapy. We calculated the mutant allele frequency for each mutation in each cell. We only retained mutations which were: (i) the difference of mean mutant allele frequencies were larger than 20%; (ii) the *p* value using student *t*-test between cells before and after radiation therapy was less than 0.05. In this way, two mutations were retained. The two mutations were: L1231F in *AKAP6* and G430E in *NFKB1* (Figure 1). Only G430E in *NFKB1* was predicted to be damaging by both SIFT [19] and PolyPhen2 [20]. Interestingly, *NFKB1* was reported to be involved in inflammation-associated cancer [21], while HPV has been linked to chronic inflammation [22]. Considering that this tumour patient was persistent infected with HPV, the somatic mutation in *NFKB1* gene was very likely to cause the radiotherapy resistance in this study.

We then focused on the function of the *NFKB1* gene and its mutation G430E. The mean mutant allele frequency for G430E in *NFKB1* in cancer cells before radiotherapy was only 9.8%, while the mean mutant allele frequency in cancer cells after radiotherapy was 31.9% (*P* = 0.048, Figure 1). The most probable explanation for this phenomenon is the tumour tissue before radiotherapy was of polyclonal origin, and the cancer cells harboring G430E only accounted for a small percentage (about 9.8% ^*^ 2 = 19.6%). When receiving radiotherapy, G430E may cause tumour resistant to radiotherapy. Thus, the cells harboring G430E have higher survival rate, and the proportion of cancer cells harboring G430E significantly increased after radiotherapy.

### NFKB1 is a tumour suppressor in cervical tumour

**Figure 2:**
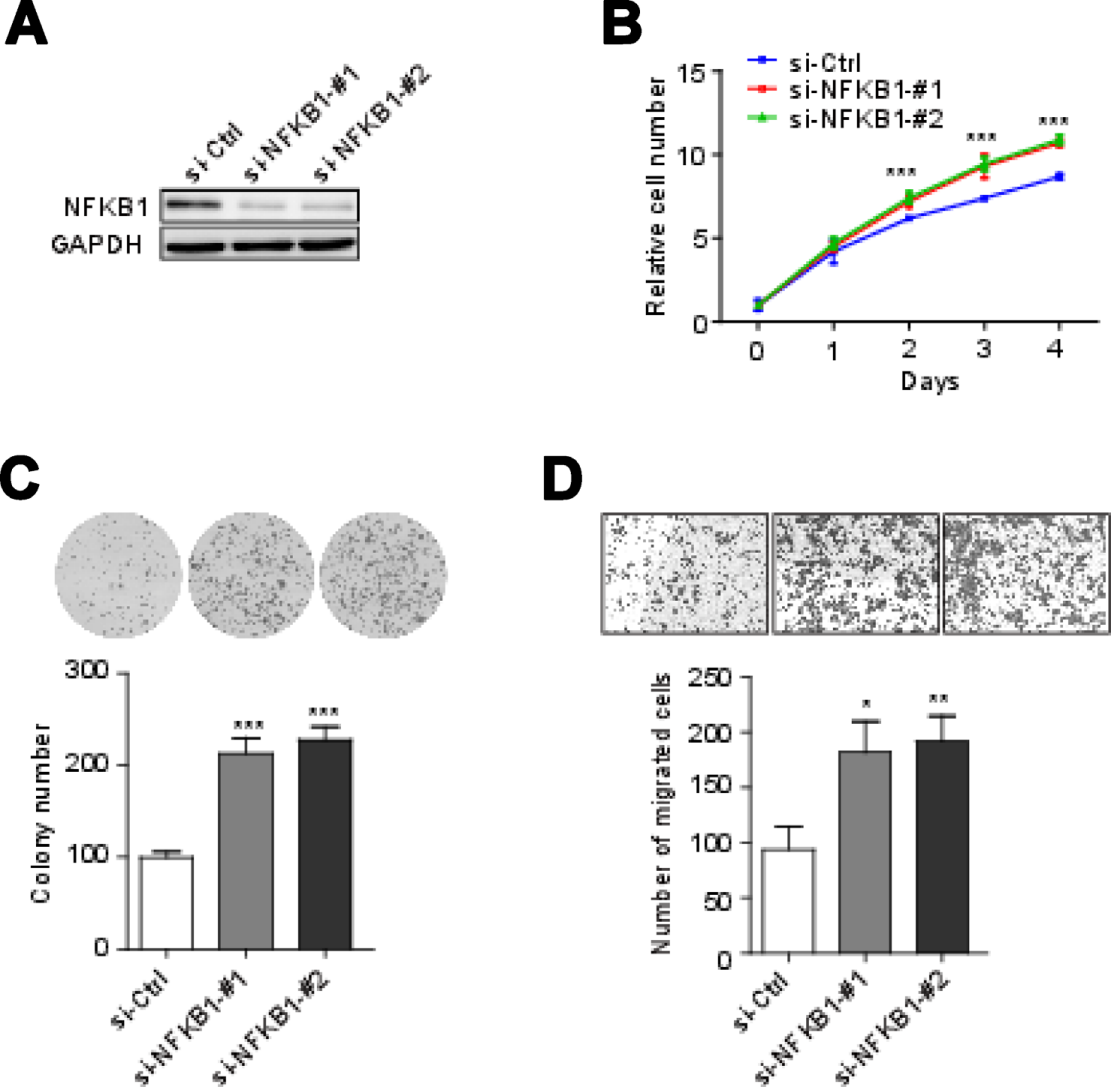
NFKB1 knockdown promoter cell growth, colony formation and migration (**A**) Effects of siRNAs targeting NFKB1 (si-NFKB1 -#1 and si-NFKB1-#2) and non-targeting scrambled siRNA controls (si-Ctrl) in Hela cells were shown by Western blotting. (**B**) Proliferation of Hela cells transient transfected with siRNAs targeting NFKB1 and non-targeting scrambled siRNA controls. (**C**) Colony formation of Hela cells transient transfected with siRNAs targeting NFKB1 and non-targeting scrambled siRNA controls. (**D**) Migration of Hela cells transient transfected with siRNAs targeting NFKB1 and non-targeting scrambled siRNA controls. Data shown as the mean ± s.d. are representative of three independent experiments performed in triplicate. ^*^P < 0.05, ^**^P < 0.01 and ^***^P < 0.001 from two-tailed unpaired *t*-tests. s.d., standard deviation.

**Figure 3:**
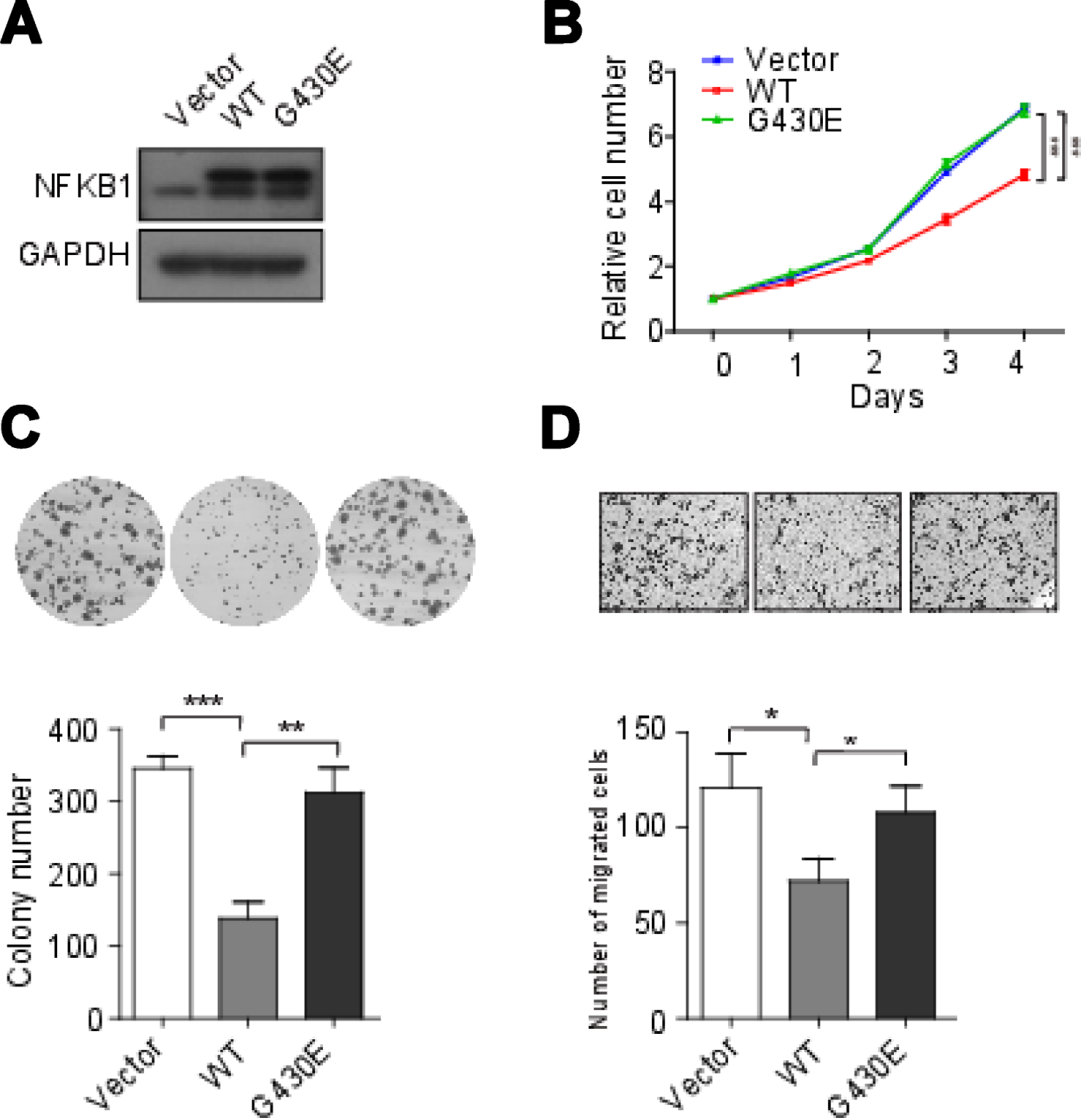
NFKB1 overexpression inhibited cell growth, colony formation and migration of Hela cells (HPV-18 positive) (**A**) Hela cells transient transfected with wild-type (WT) and mutant (G430E) NFKB1 and control vector (Vector). The protein expressions of NFKB1 were detected by Western blotting. (**B**) Proliferation of Hela cells transient transfected with WT, G430E and Vector. (**C**) Colony formation of Hela cells transient transfected with WT, G430E and Vector. (**D**) Migration of Hela cells transient transfected with WT, G430E and Vector. Data shown as the mean ± s.d. are representative of three independent experiments performed in triplicate. ^*^P < 0.05, ^**^P < 0.01 and ^***^P < 0.001 from two-tailed unpaired *t*-tests. s.d., standard deviation.

**Figure 4:**
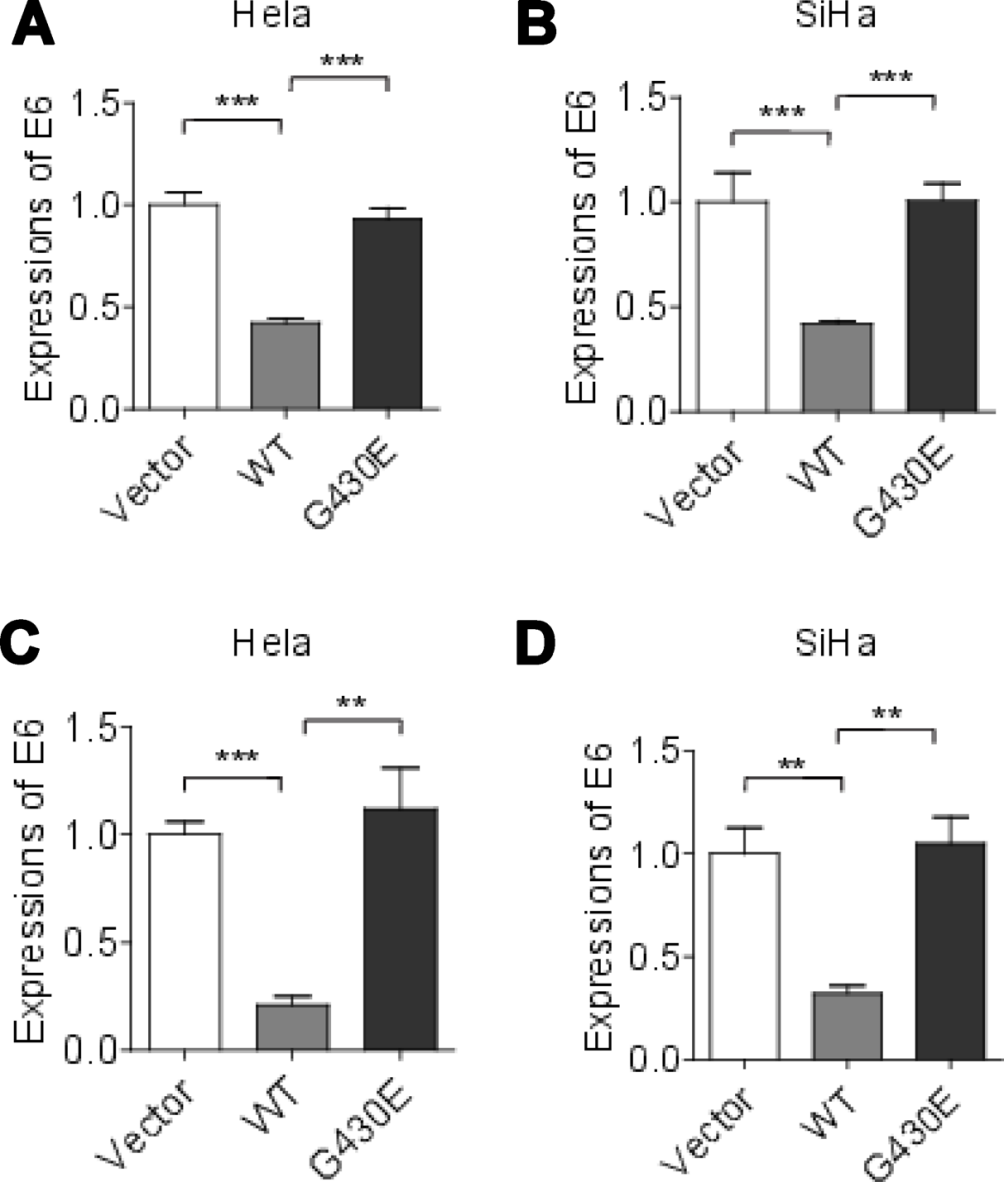
HPV E6 mRNA expressions were increased in cervical cancer cells with NFKB1 mutation (**A**) HPV-18 E6 mRNA expressions in Hela cells with wild-type (WT) and mutant (G430E) NFKB1 and control vector (Vector). (**B**) HPV-16 E6 mRNA expressions in SiHa cells with wild-type (WT) and mutant (G430E) NFKB1 and control vector (Vector). (**C**) HPV-18 E6 mRNA expressions in Hela cells with wild-type (WT) and mutant (G430E) NFKB1 and control vector (Vector) with 2 Gy of irradiation. (**D**) HPV-16 E6 mRNA expressions in SiHa cells with wild-type (WT) and mutant (G430E) NFKB1 and control vector (Vector) with 2 Gy of irradiation. Data shown as the mean ± s.d. are representative of three independent experiments performed in triplicate. ^*^P < 0.05, ^**^P < 0.01 and ^***^P < 0.001 from two-tailed unpaired *t*-tests. s.d., standard deviation.

To examine the effect of *NFKB1* gene on cervical cells, we knockdown of *NFKB1* by siRNAs (siRNA#1 and siRNA#2) in Hela cells (HPV-18 positive-cell). We confirmed that the NFKB1 expression level was significantly decreased by Western blot analysis (Figure 2A). Without radiation, cell counting kit-8 (CCK-8) assays showed that knockdown of *NFKB1* significantly increased the rate of cell growth and colony formation, as compared with the negative control (Figure 2B and 2C). Moreover, without radiation, knockdown of *NFKB1* also significantly increased cell migration, as compared with the negative control (Figure 2D). We also overexpression of wild-type *NFKB1* in Hela cells, and confirmed that the NFKB1 expression level was significantly increased by Western blot analysis (Figure 3A). Consistent with the findings of *NFKB1* knockdown, without radiation, overexpression of *NFKB1* significantly decreased the rate of cell growth, colony formation and migration (Figure 3B–3D). To check this effect in HPV-16 positive cervical cancer cells, we used SiHa cells (HPV-16 positive-cell). Similar results were observed in SiHa cells (Supplementary Figure 1). HPV E6 mRNA expression might be predictive of disease progression and might constitute a useful tool for screening or patient management [23]. We found that overexpression of *NFKB1* can also significantly decrease the mRNA expression level of HPV-18 or HPV-16 E6 in both two cell lines (Figure 4A and 4B). These results indicated that NFKB1 is a tumor suppressor in cervical tumor.

### NFKB1 mutation could weaken the tumour suppressing functions of NFKB1

To check the function of somatic mutation (G430E) in NFKB1, the mutant NFKB1 was transfected into Hela cells (HPV-18 positive-cell). Compared with wild-type NFKB1, mutant NFKB1 showed significantly increased rate of cell growth, colony formation and migration (Figure 3B–3C). Similar results were observed in SiHa cells (HPV-16 positive-cell) (Supplementary Figure 1). Moreover, we also found that mutant NFKB1 showed significantly increased mRNA expression level of E6 in both two cell lines (Figure 4A and 4B). These results suggested that the mutation G430E in NFKB1 could weaken the tumor suppressing functions of NFKB1 in cervical tumor.

### NFKB1 mutation promotes the survival of cervical cancer cells following irradiation

**Figure 5:**
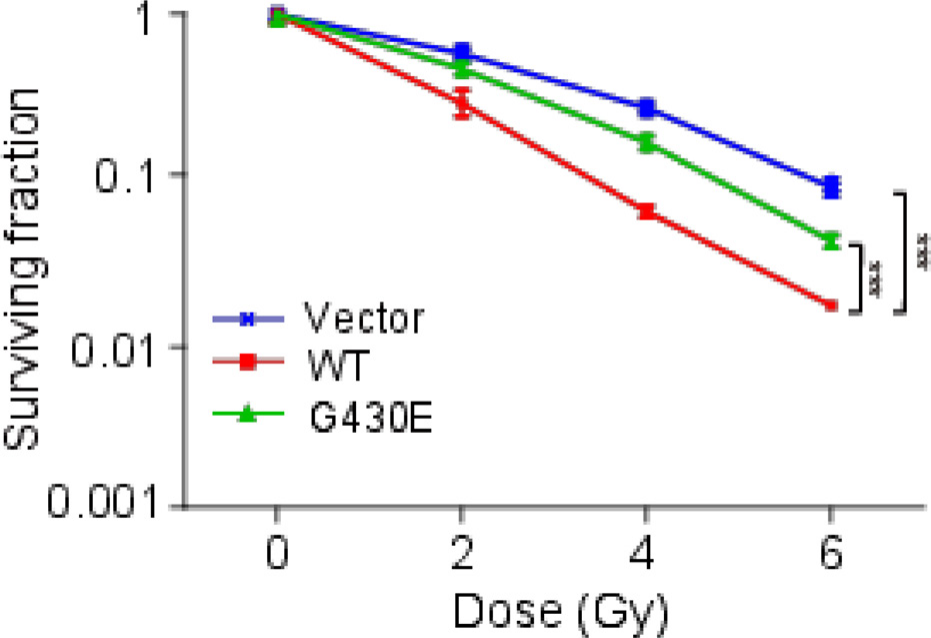
NFKB1 mutation promoted the survival of Hela cells (HPV-18 positive) following irradiation Colony formation of Hela cells with 0, 2, 4, 6 Gy of irradiation. Hela cells were transient transfected with wild-type (WT) and mutant (G430E) NFKB1 and control vector (Vector). Data shown as the mean ± s.d. are representative of three independent experiments performed in triplicate. ^*^P < 0.05, ^**^P < 0.01 and ^***^P < 0.001 from two-tailed unpaired *t*-tests. We compared WT with Vector to check the effect of NFKB1 on cervical cancer cells with radiation. We compared G430E with WT to check the effect of mutation in NFKB1 on cervical cancer cells with radiation. s.d., standard deviation.

To further investigate the effect of NFKB1 and NFKB1 mutation G430E on cervical cancer cells with radiation, we overexpression wild-type and mutant (G430E) NFKB1 in Hela cells (HPV-18 positive-cell), and the cells were then irradiated. We performed colony formation assays. Compared with empty vector, overexpression of wild-type NFKB1 further inhibit the survival of cervical cancer cells with irradiation (Figure 5). While compared with wild-type NFKB1, mutant NFKB1 (G430E) promoted the survival of cervical cancer cells with irradiation (Figure 5). We observed similar results for mutant NFKB1 (G430E) in SiHa cells (HPV-16 positive-cell) (Supplementary Figure 2). Moreover, we also found that mutant NFKB1 showed significantly increased mRNA expression level of E6 in both two cell lines with radiation (Figure 4C and 4D). These results suggested that NFKB1 mutation (G430E) promotes the survival of cervical cancer cells following irradiation, which was consistent with the result that mutation rates of this mutation are significantly elevated in cells after radiotherapy.

## DISCUSSION

In this study, we carried out single cell sequencing in 25 cells from cervical tumour samples before and after radiotherapy. To the best of our knowledge, this is the first detailed genetic landscape of a tumour before and after radiotherapy at the single cell level. We identified a missense mutation in NFKB1 (G430E), which showed significantly elevated mutation rates in cells after radiotherapy. Further functional assays showed that NFKB1 was a tumour suppressor in cervical cancer, and the mutation in NFKB1 could weaken the tumour suppressing functions of NFKB1. We also found that overexpression of NFKB1 enhanced the sensitivity of cervical cancer cells to the effects of irradiation, and the mutation in NFKB1 weakened this effect. These results suggested that the NFKB1 and the mutation in NFKB1 may be responsible for the radiotherapy resistance in cervical cancer.

Different cells from the same tumour were known to harbour different genomes. Thus, single-cell genomic analyses will help to uncover many new and longstanding questions of tumour studies [24]. For example, cell lineage relationships, tumour heterogeneity and so on. Although the rapid development of next-generation sequencing (NGS) approaches, single-cell genomic analyses was still a challenge until recent newly developed methods [24]. Wang *et al.* developed a robust MDA-based single-cell sequencing method, and carried out single cell exome sequencing of one myeloproliferative neoplasm, which revealed that this neoplasm represented a monoclonal evolution [25]. Using the same method, they performed single cell exome sequencing of one renal cell carcinoma, and demonstrated this tumour may be more genetically complex than previously thought [18]. In addition, Navin *et al.* also successfully apply single-nucleus sequencing to investigate tumour population structure and evolution in two human breast cancer cases [17]. These findings may be helpful for developing more effective cellular targeted therapies.

NFKB1, also known as p105 or p50, is one of the nuclear factors of kappa light polypeptide gene enhancer in B-cells (NF-κB) transcription factor family members. NF-κB proteins play a central and subunit-specific role in the response to DNA damage. Previous work identified NF-κB1 acts as a tumour suppressor in the setting of alkylation damage [26]. Moreover, NFKB1 was reported to be involved in inflammation-associated cancer [21]. Nfkb1 function can be seen in mouse models, where Nfkb1 ^–/–^mice display increased inflammation and susceptibility to certain forms of DNA damage, leading to cancer [27]. Mechanically, evidence also showed that NFKB1 was a negative regulator of the NF-κB, which could upregulate expression of anti-apoptotic genes, such as IAPs, cell-cycle promoters, and growth factors and their receptors [28], further supporting its tumour suppressive role. As for the mutations in NFKB1, the mutant Nfkb1^S340A/S340A^ knock-in mouse had a similar phenotype to that of the Nfkb1-/- [27]. However, in numerous human tumour tissues, NF-κB1 is overexpressed in tumor tissues, which need further studies. We further checked the data from cBioPortal, and found two of 309 cervical cancer cases harbor mutations in *NFKB1*. Moreover, mutations in *NFKB1* were also detected in various tumours (Supplementary Figure 3), suggesting that *NFKB1* play an important role in cancer. Taken together, it was reasonable that NFKB1 and its mutations play an important role in cervical cancer, which was caused by HPV infection.

Another gene of note in this study is AKAP6. The mutations in this gene (L1231F) was predicted to be damaging by SIFT [19], but benign by PolyPhen2 [20]. This gene encodes a member of the A-kinase anchor proteins (AKAPs). These proteins have the common function of binding to the regulatory subunit of protein kinase A (PKA) and confining the holoenzyme to discrete locations within the cell [29]. The previous review have delineated a role for AKAPs and their associated proteins in disease progression [29]. Thus, AKAP complexes may be proved to be useful therapeutic targets.

In summary, NFKB1 plays important roles in the regulation of tumorigenesis and malignant progression in cervical cancer. NFKB1 could enhance the sensitivity of cervical cancer cells to the irradiation, and the mutation in NFKB1 weakened this effect. Thus, NFKB1 and its mutation may be a potential molecular target in cervical cancer radiation therapy in the future.

## MATERIALS AND METHODS

### Sample collection and preparation of cell suspensions

Fresh tumour and blood samples were obtained from a 46-year-old female patient with the exogenous type of cervical carcinogenesis at Beijing Obstetrics and Gynecology Hospital. The diagnosis of cervical carcinogenesis has been described in detail previously [30]. This patient was persistent infected with HPV. The tumour was classified as IIA2 according to the 2009 International Federation of Gynecology and Obstetrics staging system. The primary tumour size was 5 cm. The patient received radiation therapy (10 Gy). However, we did not observe an improvement in the conditions of this patient. We obtained tumour tissues before and after radiotherapy. The tumour tissues were pathologically confirmed as malignant cervical carcinogenesis with more than 90% tumour cells. This study was performed with the approval of the Beijing Obstetrics and Gynecology Hospital. Signed written consent was obtained before recruitment for the study.

### Whole genome sequencing

Paired-end library preparation was conducted using Illumina protocols. Genomic DNA (400 ng) was fragmented to an insert size of ∼400 bp with a Covaris device, and size selection was performed using agarose gel excision. Deep sequencing was carried out with Illumina X10 instruments. Each sample achieved ∼38× genomic coverage.

### Somatic mutation detection

After removal of adapters and low quality reads, all sequencing reads were mapped to the human genome (hg19 build) using Burrows–Wheeler Aligner (v0.5.9) with default parameters. Somatic single nucleotide variants (SNVs) were detected by VarScan (v2.3.9). A candidate somatic mutation was called if the following criteria were met. (i) The somatic *P*-values of the variants were smaller than 0.05. (ii) Mutant allele frequencies in tumours were larger than 15%. (iii) Mutant allele frequencies in the normal control were less than 0.5%. (iv) Reads with a mutant allele were larger than 4. (v) The forward reference (fr) count (i.e. the number of forward reads that match the reference base at the locus), the reverse reference (rr) count (i.e. the number of reverse reads that match the reference base at the locus), the forward non-reference (fnr) count (i.e. the number of forward reads that do not match the reference base at the locus), and the reverse non-reference (rnf) count (i.e. the number of reverse reads that do not match the reference base at the locus) in the tumour must be 1 or greater. (vi) Only mutations detected in four samples were retained. To eliminate common germline variants, SNVs observed in dbSNP135 or the 1000 Genomes Project March 2012 data release project were excluded. Annotation was performed using snpEff (v4.2) [31]. We used GRCh37.75 for transcript identification and to determine amino acid changes. All putative somatic mutations in coding regions were validated visually. To avoid variation calling biases in single cell sequencing, we only retained mutations that were detected in at least four samples.

### Mutant allele frequency calculation

We calculated mutant allele frequency for each mutation in each cell. Thus, each mutation has 25 mutant allele frequencies. If the sequencing depth for one mutation in one cell is less than 8 X, we didn’t use this mutant allele frequency for further analyses.

### Cell transfection and cell culture

Human Hela cell (HPV-18 positive) was maintained in our lab. Human SiHa cell (HPV-16 positive) was purchased from National Infrastructure of Cell Line Resource. The Hela cells were plated and cultured in assay medium (DMEM + 10% FBS) in a humidified incubator (37°C and 5% CO_2_). The SiHa cells were plated and cultured in assay medium (DMEM with high glucose (4.5 g/L) + 10% FBS) in a humidified incubator (37°C and 5% CO_2_). For NFKB1 knockdown, the cells were transfected with siRNAs as instructed by the manufacturer.

The siRNAs targeting NFKB1 were s9504 and s9505 (Silencer Select). A non-targeting scrambled siRNA (Si-Ctrl, 5′-UUCUCCGAACGUGUCACGUTT-3′) was used as control. For NFKB1 overexpression (wild-type and mutant G430E), the cDNAs of NFKB1 for wild-type and mutant variants (G430E) were subcloned into the pLV-EGFP vectors (Stratagene, La Jolla, CA), according to manufacturer’s instruction. For NFKB1 knockdown or overexpression, the siRNAs targeting NFKB1 or lentiviral vector plasmids pLV-EGFP were transiently transfected into cells using Lipofectamine 3000 reagent according to the manufacturer’s instructions. To confirm *NFKB1* gene knockdown and overexpression, we used Western blotting assays.

### Western blotting assays

To confirm *NFKB1* gene knockdown and overexpression, we used Western blotting assays. Cells were lysed with ice-cold lysis buffer (20 mmol/L Tris-HCl, pH 7.5, 150 mmol/L NaCl, 1 mmol/L Na2EDTA, 1 mmol/L EGTA, 1% Triton, 2.5 mmol/L sodium pyrophosphate, 1 mmol/L β-glycerophosphate, 1 mmol/L Na3VO4, 1 μg/mL leupeptin, and protease inhibitor cocktail) for 30 minutes (min) in ice. Cell lysates were then collected after centrifugation at 12,000 rpm for 5 min at 4°C. Sixty micrograms of lysate protein were loaded and total cellular protein was separated with 15% SDS-PAGE and then transblotted overnight at 4°C onto Hybond-P polyvinylidene difluoride membrane (Amersham Biosciences). The membrane was probed with indicated primary antibodies at room temperature for 1 hour (h) and then washed three times with 0.1% Tween 20-TBS and incubated in a horseradish peroxidase-linked secondary antibody for 1 h at room temperature. The membrane was washed three times with 0.1% Tween 20-TBS and the immunoreactive bands were detected by using enhanced chemiluminescent plus reagent kit. The primary antibody was used: anti- NFKB1 (1:5000, proteintech, 23576-1-AP) and anti-GAPDH (1:2,000, Beijing ComWin Biotech Co.,Ltd., CW0100A).

### Cell proliferation, colony formation and migration assays

For the proliferation assays, cell proliferation was evaluated by the Cell Counting Kit-8 (Dojindo) according to the manufacturer’s instructions, and 2000 cells were plated in triplicate in 24-well dishes. For the colony formation assays, 1,000 cells (knockdown of *NFKB1* assay) or 2,000 (overexpression of *NFKB1* assay) per well were plated onto a 10 cm plate, cultured for 14 days, and stained with crystal violet. For the cellular migration assays, 1.0 × 10^4^ cells that starved without serum for 12 h were placed in the top chamber with a non-coated membrane. Cells were starved overnight in serumfree medium, trypsinized and washed three times in DMEM containing 1% FBS. Cells were then suspended in 500 µL of DMEM containing 1% FBS and added to the upper chamber, while 500 µL of DMEM containing 10% FBS was placed in the lower chamber. After 48 h of incubation, the matrigel and the cells remaining in the upper chamber were removed with cotton swabs. The cells on the lower surface of the membrane were fixed in methanol and stained with 0.5% crystal violet. All experiments were carried out in triplicates. For the radiosensitivity assay, the radiosensitivity assay was conducted, and colony formation was used to measure the effect of NFKB1 and its mutation on radiation sensitivity. 1000, 4000, 1.0 × 10^4^ or 1.0 × 10^5^ cells per well were plated onto a 10 cm plate with radiation (0, 2, 4 or 6 Gy).

### HPV E6 mRNA based RT-PCR

Total RNA from HeLa cells and SiHa cells were extracted using Trizol. The HPV E6 expressions were detected using RT-PCR. The primers used were listed: HPV-18 E6: 5′-CCAGAAACCGTTGAATCCAG-3′ (Forward) and 5′-GTTGGAGTCGTTCCTGTCGT-3′ (Reverse); HPV-16 E6: 5′-CAGGAGCGACCCAGAAAGTT-3′ (Forward) and 5′-GCAGTAACTGTTGCTTGCAGT-3′ (Reverse). The primers for the GAPDH control were 5′-AAGGTCGGAGTCAACGGATTT-3′ (Forward) and 5′ ACCAGAGTTAAAAGCAGCCCTG 3′ (Reverse).

Experiments were performed in triplicate in three independent experiments.

### Statistical analyses

Differences of cell number, foci number and migration between groups were analyzed using unpaired *t* test. All data are reported as means ± s.d. Statistical tests were performed using the R (v3.0.0) package. The significance level was set at *P* < 0.05.

## SUPPLEMENTARY MATERIALS FIGURES AND TABLE





## Abbreviations

NFKB1: nuclear factor-kappa beta 1
PIK3CA: phosphoinositide-3-kinase, catalytic, alpha polypeptide
TP53: Tumor protein p53
