# Supplementary material for: Single cell whole genome sequencing reveals that NFKB1 mutation affects radiotherapy sensitivity in cervical cancer
Source: Oncotarget. 2017 Dec 21;9(7):7332–40. doi: 10.18632/oncotarget.23587 (PMC5800906; doi:10.18632/oncotarget.23587)
Supplement: Supplementary file 1 [file oncotarget-09-7332-s001.pdf]

Single cell whole genome sequencing reveals that NFKB1 mutation affects radiotherapy sensitivity in cervical cancer

SUPPLEMENTARY MATERIALS

Collection of single cells, preparation of cell lysates and multiple displacement amplification

Firstly, we prepared a 200 µl PCR tube containing 1.5 ml ALB for each cell to be collected and kept the tubes on ice. We ensured that the ALB droplet is at the bottom of the tube. We prepared culture dishes with rows of 3 droplets (~3 µl) of Ca^{2+}- and Mg^{2+}-free medium and one large droplet (~20 µl). For cells that are obtained from cell suspensions, we washed cells three times in PBS. A manual-controlled pipetting system was used to transfer 1–2 µl of the cell suspension to the large droplet of Ca^{2+}- and Mg^{2+}-free medium in the culture dish. We then used this system to transfer 2–3 cells to each first droplet of the rows of three, visualizing this procedure under an inverted microscope (Nikon Instruments Co., Ltd.). We then washed a single cell by transferring it from droplet to droplet using the manual-controlled pipetting system, visualizing this procedure under the inverted microscope. We transfered the cell into the 1.5 µl ALB contained in the 200 µl PCR tubes using the manual-controlled pipetting system and keep the tube on ice. We added 1 µl of the last washing droplet to an additional PCR tube for use as a negative control. We stored the samples at –20°C for at least 30 min.

Each cell was transferred into a precooled PCR tube containing a cell lysis solution. The samples were incubated in a thermocycler for 10 min at 65°C. A physiological saline blank was included as a negative control.

Whole genome amplification (WGA) was performed using a REPLI-g Mini Kit (Qiagen GmbH) according to the manufacturer's protocol. All samples were amplified by MDA. A reaction in a total volume of 50 µl was performed at 30°C for 16 h and then terminated at 65°C for 10 min. Amplified DNA products were then stored at −20°C.

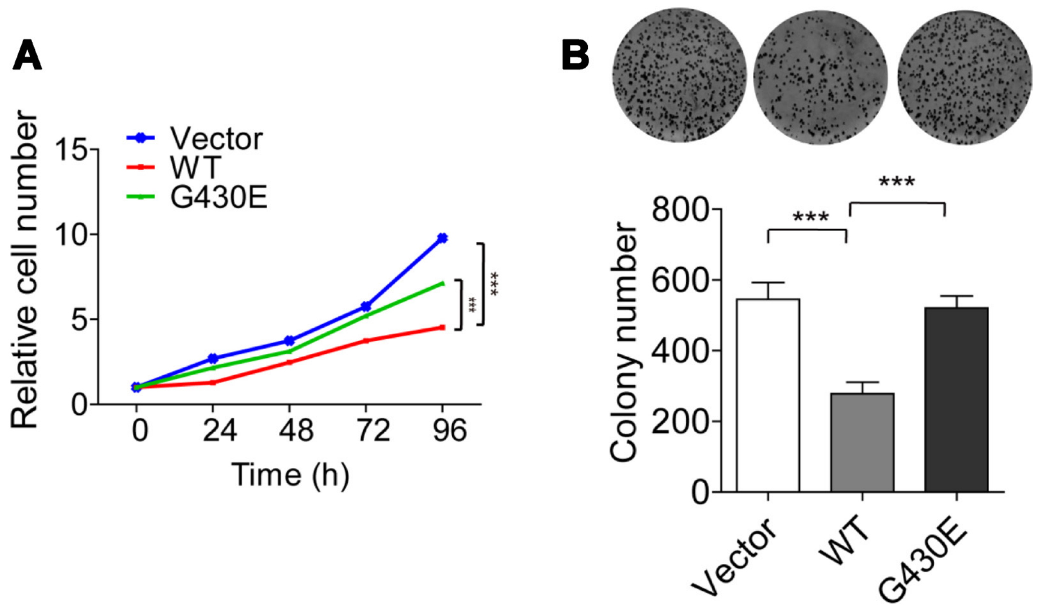

Supplementary Figure 1: NFKB1 overexpression inhibited cell growth, colony formation and migration of SiHa cells (HPV-16). (**A**) Proliferation of SiHa cells transient transfected with wild-type (WT) and mutant (G430E) NFKB1 and control vector (Vector). (**B**) Colony formation of SiHa cells transient transfected with wild-type (WT) and mutant (G430E) NFKB1 and control vector (Vector). Data shown as the mean ± s.d. are representative of three independent experiments performed in triplicate. $^{*}P < 0.05$, $^{**}P < 0.01$ and $^{***}P < 0.001$ from two-tailed unpaired *t*-tests. s.d., standard deviation.

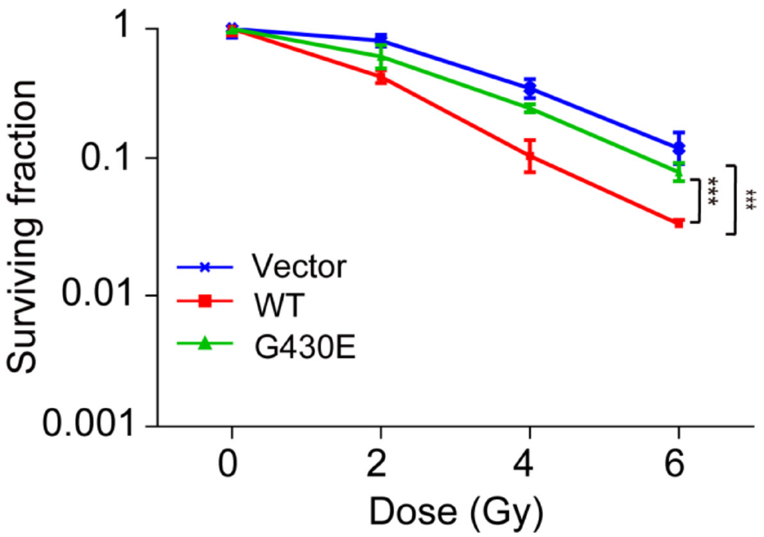

Supplementary Figure 2: NFKB1 mutation promoted the survival of SiHa cells (HPV-16 positive) following irradiation of SiHa cells. Colony formation of SiHa cells with 0, 2, 4, 6 Gy of irradiation. SiHa cells were transient transfected with wild-type (WT) and mutant (G430E) NFKB1. Data shown as the mean ± s.d. are representative of three independent experiments performed in triplicate. $^{*}P < 0.05$, $^{**}P < 0.01$ and $^{***}P < 0.001$ from two-tailed unpaired *t*-tests. s.d., standard deviation.

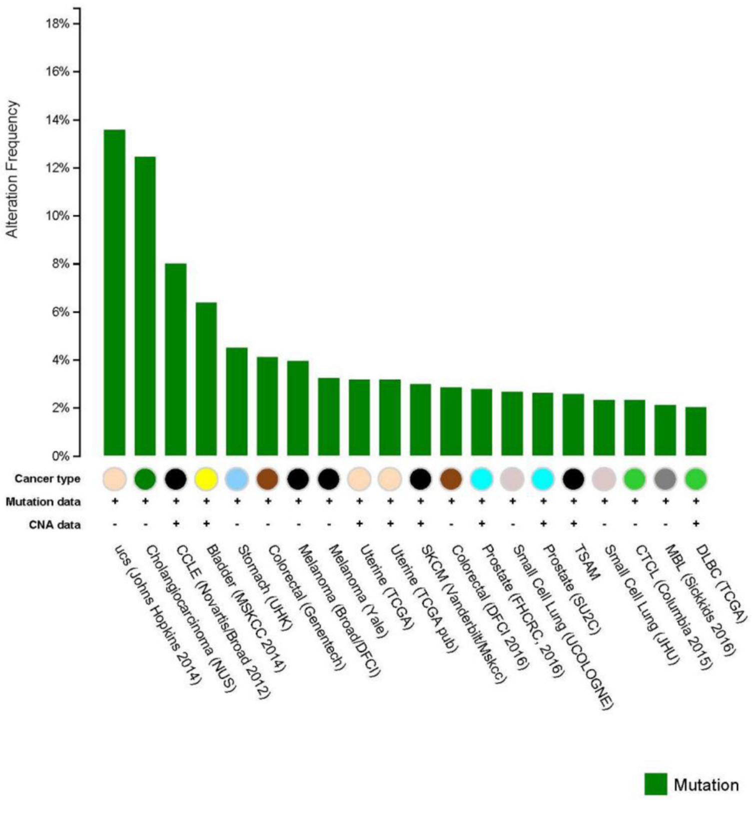

Supplementary Figure 3: *NFKB1* mutational frequencies in various cancers. The data was from cBioPortal. We only show the studies whose *NFKB1* mutational frequencies were more than 2%.

Supplementary Table 1: Mutations detected in single cell sequencing. See Supplementary_Table_1
